# Changes in the Vaginal Microbiome during the Pregnancy to Postpartum Transition

**DOI:** 10.1007/s43032-020-00438-6

**Published:** 2021-01-11

**Authors:** Kenetta L. Nunn, Steven S. Witkin, G. Maria Schneider, Allison Boester, Dimitrios Nasioudis, Evelyn Minis, Karol Gliniewicz, Larry J. Forney

**Affiliations:** 1 Institute for Bioinformatics and Evolutionary Studies, University of Idaho, Moscow, ID USA; 2 Bioinformatics and Computational Biology Graduate Program, University of Idaho, Moscow, ID USA; 3 Department of Obstetrics and Gynecology, Weill Cornell Medicine, New York, NY USA; 4 Virology Laboratory, Institute of Tropical Medicine, University of São Paulo, São Paulo, Brazil; 5 Department of Biological Sciences, University of Idaho, 875 Perimeter MS 3051, Moscow, ID 83844-3051 USA

**Keywords:** Pregnancy, Postpartum, Vagina, Vaginal microbiome

## Abstract

Substantial changes in the composition of the vaginal microbiome occur following the end of pregnancy. To identify potential drivers of microbiome changes in individual women during the pregnancy to postpartum transition, we evaluated vaginal samples from 48 pregnant women during their first and third trimesters and postpartum. We determined the species composition of vaginal communities and the vaginal fluid levels of compounds involved in mediating changes in host physiology and the immune system at each time point. We used linear mixed-effects models to characterize associations. Consistent with previous reports, but with a larger sample size, a US population, and variations in the dominant bacteria, the vaginal microbiome was found to be more diverse during the postpartum period. There was a lower abundance of *Lactobacillus* and significantly higher proportions of *Streptococcus anginosus* and *Prevotella bivia*. Moreover, we uniquely demonstrated that postpartum vaginal secretions were also altered postpartum. There were elevated levels of hyaluronan and Hsp70 and decreased levels of the D- and L-lactic acid isomers. We posit that these variations are consequences of alterations in the vagina after delivery that profoundly alter the host environment and, thus, lead to changes in the capability of different bacterial species to survive and proliferate.

**Supplementary Information:**

The online version contains supplementary material available at 10.1007/s43032-020-00438-6.

## Introduction

During a healthy pregnancy, the vaginal microbiome is characterized by low bacterial species diversity [1, 2] and is typically dominated by one of several different species of *Lactobacillus* [3]. A few cross-sectional [4] and longitudinal studies [5–7] have compared the vaginal microbiome during pregnancy and in the postpartum period. These studies confirmed that considerable changes in vaginal community composition occur immediately following pregnancy. During this transition, there is an overall decrease in *Lactobacillus* [4, 5, 7] with a concomitant increase in diverse taxa including but not limited to Clostridia (*Peptoniphilus* and *Anaerococcus*), Bacteroidia, *Prevotella*, *Veillonella*, *Porphyromonas*, and *Megasphaera* [4–7]. As expected, the proliferation of diverse taxa during the postpartum period is paralleled by increases in ⍺-diversity [4–7]. Doyle et al. obtained vaginal samples from a cohort of Malawi women anywhere from 5 to 583 days post-delivery and demonstrated that *Lactobacillus* spp. were present in less than a third of the women [8]. Furthermore, postpartum vaginal communities resembled those of community state types (CST) III and IV, as defined by Ravel et al. [8, 9]. CST-III consists of communities in which *Lactobacillus iners* is dominant. In contrast, CST-IV vaginal communities are heterogeneous in composition with low proportions of lactobacilli and elevated levels of an assortment of anaerobic bacteria. They also noted that the abundances of *L. iners* increased with time throughout the postpartum stage [8]. In comparison to other *Lactobacillus*-dominant communities, those dominated by *L. iners* tend to be more diverse and less stable [10–12] as they often transition to communities resembling CST-IV [13]. The observed shift in bacterial community composition during the postpartum period can occur as early as the onset of labor [7, 14] and may persist for up to 1 year [5].

Alterations in vaginal secretions would also be anticipated to accompany changes in the vaginal microbiome in the postpartum period. Specifically, a decrease in the levels of compounds produced chiefly by lactobacilli and increases in those associated with an elevated level of physiological stress or degradation of the extracellular matrix would be expected. Currently, only limited information exists on host-associated changes in the vaginal microbiome in individual women during pregnancy and postpartum. In the present study, we determined the species composition of vaginal communities and the levels of vaginal fluid compounds involved in host physiology and immune system modifications in pregnant women within the first and third trimesters and approximately 1 month postpartum.

## Materials and Methods

### Study Participants and Sample Collection

Our prospective study included pregnant women seen for outpatient obstetrics services at Weill Cornell Medicine in New York City. Exclusion criteria included inability to give informed consent, signs or symptoms of a gynecological disorder or infection at the time of examination, multifetal gestation, presence of an autoimmune or endocrine problem, antibiotic use in the previous 4 weeks, or vaginal bleeding. In total, 48 women were enrolled and provided vaginal samples during their first (≤ 12 weeks) and third (28–38 weeks) trimesters, and 28–45 days postpartum. All of the women in this study had vaginal deliveries. Of those 48 women, 32 provided samples for each time point. Samples from the posterior vagina were obtained with a cotton swab, vigorously shaken into a sterile tube containing 1 mL of sterile phosphate-buffered saline (PBS) and centrifuged, and aliquots of the supernatant were stored at ‑ 80 °C until analyzed. The study was approved by the Institutional Review Board at Weill Cornell Medicine. All subjects gave informed, written consent.

### Compound Measurements

Vaginal levels of the D- and L-lactic acid isomers were quantitated by colorimetric assays using the EnzyChrom D-lactic acid and L-lactic acid kits (BioAssay Systems, Haywood, CA). The levels of matrix metalloproteinase-8 (MMP-8) (R&D Systems, Minneapolis, MN), extracellular matrix metalloproteinase inducer (EMMPRIN) (R&D Systems), neutrophil gelatinase-associated lipocalin (NGAL) (R&D Systems), hyaluronan (R&D Systems), α-amylase (human pancreatic α-amylase, Abcam, Cambridge, UK), the stress-inducible 70-kDa heat shock protein (HSPA1A) commonly known as Hsp70 (R&D Systems), and p62 (Enzo Life Sciences, Farmingdale, NY) were determined using commercially available ELISA kits. Hsp70 and p62 were measured from the supernatant of lysed epithelial cells, while all of the other compounds were measured directly from vaginal secretions. To obtain the epithelial cell lysate, vaginal secretions were centrifuged, and the epithelial cell pellet was immediately lysed in a detergent containing a protease inhibitor cocktail (Sigma, St. Louis, MO). The supernatant from the lysate was stored in aliquots at ‑ 80 °C. The total protein levels in samples were measured using a colorimetric assay (Thermo-Fisher Scientific, Waltham, MA). Concentrations were determined from a standard curve that was generated in parallel to test samples and converted to μmoles per mL, units per mL, or pg per mL. When applicable, measurements were normalized by the total amount of protein in the sample and reported in pg, ng, or units per μg of total protein. All assays were performed by staff blinded to all clinical information. These compounds were chosen for analysis since each had previously been shown to be associated with alterations in the composition of the vaginal microbiota or with immunological properties of vaginal epithelial cells.

### Determining Vaginal Community Composition

The species composition of vaginal microbial communities was determined by classifying partial 16S rRNA gene sequences as previously described [15]. In brief, total genomic DNA was extracted from 250 μL of vaginal swabs stored in 1X PBS using chemical and mechanical lysis and purified using QIAamp DNA mini kits (Qiagen). Genomic DNA concentrations were determined using the Quant-iT™ PicoGreen™ dsDNA assay kit (Invitrogen). For amplicon sequencing, the V1-V3 16S rRNA gene regions were amplified using a two-step PCR protocol, first amplifying the gene region using universal primers 27F and 534R, and then adding sample barcodes and sequence adapters. Amplicons were sequenced using the Illumina MiSeq at the University of Idaho. Forward and reverse reads were paired using FLASH [16], processed through DADA2 v 1.12.1 [17] to identify distinct sequences, and the sequences were classified into genus and species level using SPINGO [18].

A total of 358 taxa were identified, and these data were used to analyze the rank abundance of taxa and measure ⍺-diversity. For subsequent analyses, including visualizing community composition, we created a smaller dataset of taxon abundance for 57 taxa. Fifty-six of the 358 taxa were present at a minimum of 1% in at least two individuals. The remaining taxa (302) were categorized as other. The corresponding relative abundance data are reported in Table S1. Samples that did not have at least 3000 sequence reads were discarded from further analysis. This resulted in 47, 45, and 34 samples from the first trimester, third trimester, and postpartum stages, respectively. Thirty-two women had all three time points, 14 women had two time points, and two women had only one time point.

### Bioinformatics and Statistical Analyses

All analyses were performed using R v 3.6.0 [19]. In addition to the base packages, the following packages were used for organizing data, performing analyses, and to produce figures: cluster [20], e1071 [21], FSA [22], ggplot2 [23], ggpubr [24], lme4 [25], lmerTest [26], multcomp [27], plyr [28], tidyr [29], and vegan [30]. To determine what kinds of communities were present in this cohort, we performed complete-linkage hierarchical clustering on alt-Gower distances computed from taxon relative abundance data. We then used silhouette information to identify ten clusters, numbered 1 through 10. Alpha diversity was measured using the Shannon and Simpson diversity indices.

Linear mixed-effects models were used to model the means of the response variables between pregnancy stages to evaluate significant differences. From this point forward, stage refers to the first and third trimesters and the postpartum period. In these models, subject was included as a random variable to account for any within-subject variation across stages. The various response variables included bacterial species abundances, measures of ⍺-diversity, and compounds measured in vaginal fluids. Statistical comparisons were performed by testing general linear hypotheses and multiple comparisons of the means using Tukey’s test. If the linear mixed-effects model resulted in a singular fit, we used a Kruskal-Wallis rank sum test to evaluate significant differences in the means of the response variables between stages. A significant Kruskal-Wallis test was followed with post hoc analysis of multiple comparisons using Dunn’s test with Bonferroni adjustment. To test associations with changes in Shannon and Simpson diversity, we created a base mixed-effects model. We included stage and vaginal compounds that differed significantly by stage as constant variables (fixed effects). To account for differences in the subject across stages, we treated the subject as a random variable. Using these base models, we performed stepwise linear regression to determine which variables to include in the final models. A summary of each final model was generated to determine associations. The significance threshold was set to *p* < 0.05.

## Results

The characteristics of the 48 women enrolled in the study are shown in Table 1. Their mean age was 33.4 years, and the mean body mass index was 22.3 kg/m^2^. All delivered healthy infants at term at a mean gestational age of 39.5 weeks. This was the first pregnancy for 15 (31.3%) of the women, while 33 (68.8%) experienced a prior delivery. The majority of subjects were white. We found that all subjects had a vaginal pH < 4.5 (data not shown).

**Table 1:** Characteristics of study participants

Characteristic	No. of women or %	Mean	SD
Age at delivery (year)	48	33.4	3.6
Body mass index (kg/m^2^)	44	22.3	2.6
Weeks of gestation	48	39.5	1.0
Race	48		
White	56.3%		
Black	2.1%		
Asian	12.5%		
Hispanic	4.2%		
Other or mixed	25.0%		
Gravida	48	2.2	1.1
Primiparous	15		
Prior term births	23		
Prior preterm births	2		
Prior spontaneous miscarriages	11		

*SD*, standard deviation

### Comparison of the Vaginal Microbiome from Pregnancy to Postpartum

**Fig. 1:**
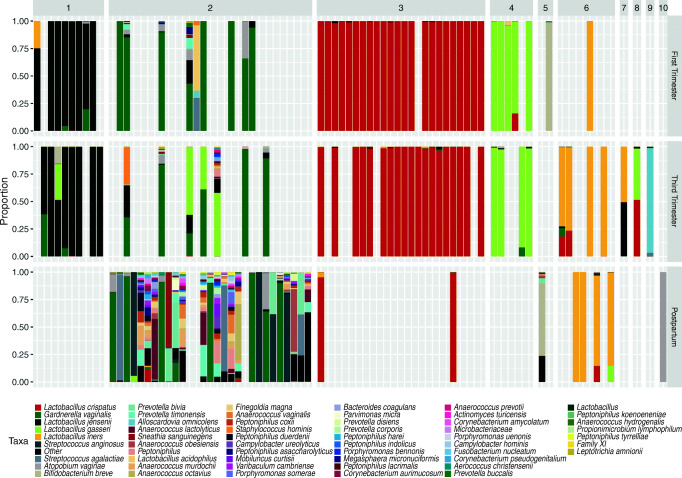
Relative proportions of bacteria in the vaginal communities of 48 pregnant women separated by stage and cluster. The stacked bars represent the proportions of bacterial taxa within one sample. Bars are separated by the pregnancy stage in which the sample was collected (right heading), and the cluster the sample was grouped into (top heading). Taxa colors are indicated in the legend below the figure. “Other” represents the sum of all bacterial taxa that were not present at 1% or more in at least two women

The relative proportions of 57 taxa identified in each stage of pregnancy are shown in Fig. S1. Vaginal communities during the first and third trimesters were mostly dominated by *Lactobacillus* species, whereas postpartum communities had low proportions of lactobacilli and were more diverse. To get a better sense of the kinds of communities that were present at each stage, we grouped relative abundance data based on similarities and differences using complete-linkage hierarchical clustering. In total, we identified ten clusters of community types. Figure 1 shows a stacked bar chart of samples separated by cluster and pregnancy stage. Vaginal communities in the first and third trimesters tended to be very similar. Roughly, 79% of the first trimester samples and 78% of the third trimester samples had vaginal communities in which *Lactobacillus* spp. were dominant. In contrast, postpartum vaginal communities were much more heterogeneous in composition. Approximately 77% of the postpartum vaginal communities had low proportions of *Lactobacillus* spp. and a diverse array of bacteria, including *G. vaginalis*, *Prevotella* spp., *Streptococcus* spp., and numerous others. At each stage, there appeared to be no difference in the composition of vaginal communities between primiparous and multiparous women (data not shown).

**Fig. 2:**
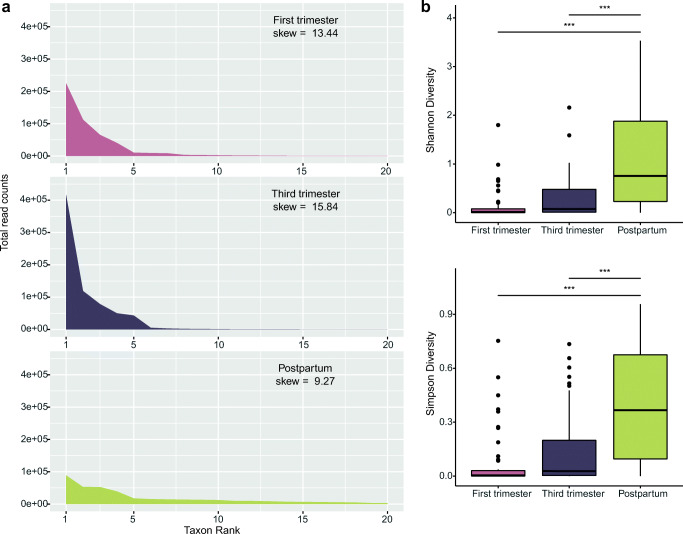
Rank abundance and ⍺-diversity profiles of samples collected during the first trimester, third trimester, and postpartum. Panel **a** shows the counts of the top-ranked taxa among first trimester, third trimester, and postpartum samples. Panel **b** shows the distribution of Shannon and Simpson diversity measures for each stage. The thick band within the box represents the median for the data, and the lower and upper boxes represent the 25th and 75th quartiles, respectively. The upper and lower whiskers represent the 95% confidence interval for the median. Individual dots above represent outliers that were not removed. Statistical significance is indicated above the lines (“***” = *p* < 0.001)

To visualize the distribution of taxa and compare diversity between pregnancy stages, we prepared rank abundance profiles for the top 20 taxa in each stage and measured the skewness in these data (Fig. 2a). Skewness measures the degree to which a sample distribution varies from normal. Compositional microbiome data typically have a long-tailed distribution and are positively skewed. This results from communities having few species that are highly abundant and many that are present at low abundances. Third trimester samples had the highest skew (15.84) followed by first trimester (13.44) and postpartum samples (9.27). This suggests postpartum samples had a distribution of taxa that were more even than the first and third trimester samples.

Next, we measured ⍺-diversity using the Shannon and Simpson diversity indices and used linear mixed-effects models to determine whether ⍺-diversity differed between stages (Fig. 2b). The Shannon diversity increased with each stage. Likewise, there were 44 species found in first trimester samples, 112 in third trimester samples, and 225 in postpartum samples. Measures of Simpson diversity followed a similar trend. The increase in ⍺-diversity from early to late pregnancy was not significant. However, ⍺-diversity was significantly higher in postpartum vaginal communities when compared to those of the first (*p* < 0.001; Shannon and Simpson diversity) and third trimesters (*p* < 0.001; Shannon and Simpson diversity).

### Changes in Numerically Abundant Taxa from Pregnancy to Postpartum

**Fig. 3:**
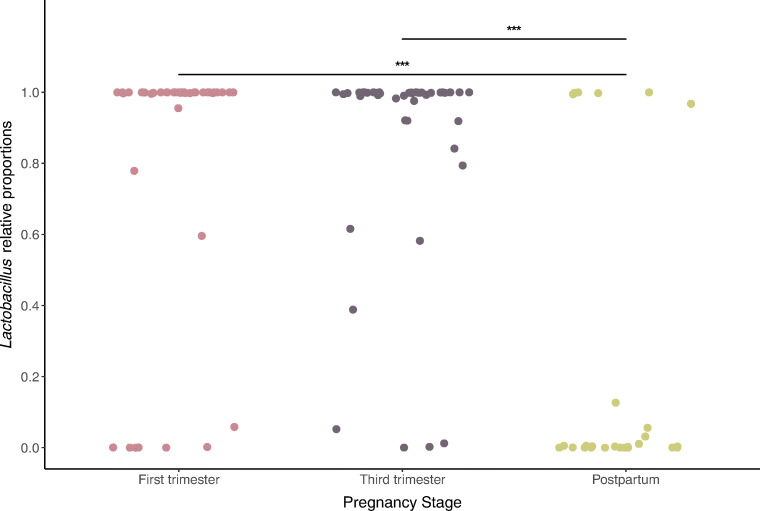
Differences in the relative abundances of *Lactobacillus* in the first and third trimesters, and postpartum. Each dot is one sample from a woman. Statistical significance is indicated above the lines (“***” = *p* < 0.001)

To quantify the change in *Lactobacillus* during pregnancy and afterward, we used linear mixed-effects models to model the means of *Lactobacillus* relative proportions between stages and controlled for within-subject variation. The distribution of the relative abundances of *Lactobacillus* by stage is shown in Fig. 3. There was no change in the mean relative abundance of *Lactobacillus* over gestation. However, we observed a sharp decrease in the abundances of lactobacilli among vaginal communities sampled during the postpartum stage in comparison to those sampled during the first and third trimesters (*p* < 0.01). Next, we evaluated whether there were significant shifts in individual lactobacilli and other top-ranked taxa in the transition from pregnancy to the postpartum period (Table 2, Table S2). We found postpartum vaginal communities to have significantly lower abundances of *L. crispatus*, *L. jensenii*, and *L gasseri*, but not *L. iners*. Instead, vaginal communities sampled during the postpartum period had markedly higher proportions of *Streptococcus anginosus* and *Prevotella bivia*. *Streptococcus agalactiae* was present at higher abundances in a few postpartum samples, but overall, these differences were not significant. Likewise, there was relatively no change in *G. vaginalis*, *Atopobium vaginae*, or *Bifidobacterium breve* across stages.

**Table 2:** Mean relative proportions for the most abundant taxa in each stage of pregnancy. The following ten taxa below were identified by taking the top ten ranked taxa for each stage, collapsing them together, and keeping only those greater than 1% in all samples

	Mean relative proportions			*p* values for comparison of the means^b^		
Taxa	First Trimester (*N*^a^ = 47)	Third Trimester (*N* = 45)	Postpartum (*N* = 34)	*p* value^c^	First - Postpartum^d^	Third - Postpartum^d^
*Lactobacillus crispatus*	49.1	44.0	6.7	*0.00*	***	***
*Gardnerella vaginalis*	15.0	10.0	18.6	0.29		
*Lactobacillus jensenii*	16.2	17.4	0.0	*0.00*	**	***
*Lactobacillus gasseri*	10.2	13.9	0.6	*0.01*	·	**
*Lactobacillus iners*	2.7	8.9	11.0	0.21		
*Streptococcus anginosus*	0.0	0.0	10.1	*0.00*	***	***
*Streptococcus agalactiae*	0.6	0.1	6.2	0.11		
*Atopobium vaginae*	1.2	0.3	4.6	0.35		
*Bifidobacterium breve*	2.1	0.4	2.0	0.69		
*Prevotella bivia*	0.0	0.0	4.6	*0.00*	***	***

^a^*N* = the number of samples/observations in each category

^b^The asterisks correspond to the following levels of significance for the *p* values: ‘.’, *p* < 0.1; *, *p* < 0.05; **, *p* < 0.01, ***, *p* < 0.001

^c^The p values in this column result from running linear mixed-effects models or Kruskal-Wallis tests to test for significant differences between the means of each stage; *p* values in italics are significant

^d^The *p* values in this column result from evaluating multiple comparisons between group means using Tukey’s test for linear models or the Dunn’s test with a Bonferroni adjustment

Next, we compared the number of women that transitioned to and from a community that was dominated by *Lactobacillus* spp. Therefore, we identified the dominant bacterial species in the vaginal communities at each stage sampled (Table S3). Here, dominance was defined as having a relative proportion higher than 50%. A community was labeled as “mixed” if it contained a more even representation of taxa and was not dominated by any one species. Among the three stages, *Lactobacillus* was dominant in 35, 38, and six communities of the first trimester, third trimester, and postpartum samples, respectively. To assess transitions to and from a *Lactobacillus*-dominant community, we only considered the 32 women for which samples were available for each stage. Transitions from the third trimester to postpartum occurred more frequently than from first to third trimester (Table 3). Roughly, a third (*N* = 10) of the women transitioned in community composition between the first and third trimesters, whereas 88% (*N* = 28) transitioned between the third trimester and postpartum. From early to late pregnancy, transitions to or between communities dominated by *Lactobacillus* were more common. Despite the high prevalence of *Lactobacillus* during gestation, most of the transitions that occurred between the third trimester and postpartum were from a community that was dominated by *Lactobacillus* to one that was not. Notably, there were no transitions in the opposite direction.

**Table 3:** Transitions between dominant bacteria among 32 women with samples from all three time points

Transition^a^	First ➝ Third	Third ➝ Postpartum
LB ➝ LB	4	4
LB ➝ non LB	1	22
non LB ➝ LB	4	0
non LB ➝ non LB	1	2
Total # of transitions	10	28
% transitions^b^	31	88

^a^Transitions are defined as LB for communities dominated by species of *Lactobacillus* or non LB for communities not dominated by species of *Lactobacillus*

^b^% transitions = (total number of transitions/number of women) × 100

### Analysis of Vaginal Compounds

**Fig. 4:**
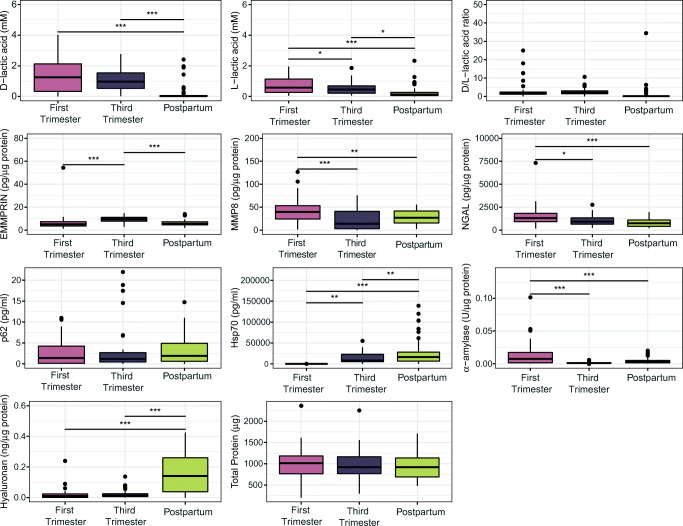
Boxplots showing the concentrations of vaginal biomarkers grouped by stage in pregnancy. The thick band within the box represents the median for the data, and the lower and upper boxes represent the 25th and 75th quartiles, respectively. The upper and lower whiskers represent the 95% confidence interval for the median. Individual dots above represent outliers that were not removed. Statistical significance is indicated above the lines as follows: “*”, *p* < 0.05; “**”, *p* < 0.01; “***” = *p* < 0.001

We sought to determine if vaginal biomarkers involved in host physiology and immunity were associated with the changes observed during the postpartum period. To do this, we assessed differences in compounds that were measured in vaginal fluids and epithelial cells using linear mixed-effects models (Fig. 4). In comparison to both early and late pregnancies, postpartum samples were characterized by elevated levels of hyaluronan (*p* < 0.001) and Hsp70 (*p* < 0.01) and lower levels of D (*p* < 0.001) and L-lactic acid (*p* < 0.001, *p* < 0.05, respectively). Next, we regressed ⍺-diversity measures against these four compounds with subject as a random effect. Using stepwise linear regression, we selected only those that were significantly associated with an increase in ⍺-diversity observed in the transition from pregnancy to the postpartum stage (Table S4). We found that an increase in ⍺-diversity was negatively associated with levels of L-lactic acid (*p* < 0.05, Shannon diversity; *p* < 0.001, Simpson diversity), but positively associated with hyaluronan (*p* < 0.001, for both), and the postpartum stage (*p* < 0.01, Shannon diversity).

## Discussion

We demonstrated that in the transition from pregnancy to the postpartum period, there was a sharp decline in the abundances of three *Lactobacillus* spp., namely *L. crispatus*, *L. gasseri*, and *L. jensenii*. This coincided with an increase in the relative abundances of other diverse bacterial taxa, while the levels of *L. iners* remained largely unchanged. These findings are consistent with previous studies that found the composition of vaginal communities to change considerably from pregnancy to the postpartum stage [5–7]. The majority of the women in our study that had communities dominated by vaginal lactobacilli during late pregnancy transitioned to communities with low proportions of lactobacilli during the postpartum stage. Similar results were reported in a study that surveyed European women during their gestation to 6 weeks after birth. MacIntyre et al. reported that 60% of the postpartum communities shifted to CST-IV from a community previously dominated by *Lactobacillus*. In that study, the transition in community composition following pregnancy was shown in a small number of samples (*N* = 15). To our knowledge, the present study is the first to be performed on pregnant women in the USA, contained a larger cohort (*N* = 32), and differed in the dominant bacterial species observed. In addition to the bacterial changes, we were able to uniquely identify an increase in the levels of hyaluronan and Hsp70 and a decrease in the levels of D- and L-lactic acid in the pregnancy to postpartum transition. These observations strongly reinforce that *Lactobacillus*-dominant communities, while relatively stable during pregnancy [31], and the levels of compounds in vaginal secretions, are rapidly altered following delivery.

The abundance of *Lactobacillus* in the vagina has been positively associated with the level of vaginal estrogen (reviewed in [32, 33]). Thus, the decrease in the abundance of *Lactobacillus* may well be the result of reduced levels of estrogen post-delivery (reviewed in [34]). During pregnancy, the placenta produces two forms of estrogen, namely estradiol and estriol, and this causes a sharp increase in estrogen levels with estradiol and estriol increasing two to three orders of magnitude, respectively (reviewed in [34]). This is paralleled by higher abundances of *Lactobacillus* during pregnancy (reviewed in [35]). Once the placenta is removed after birth, estrogen levels fall precipitously (reviewed in [34]). However, it is unclear exactly how much the overall levels of estrogen change. One study reported serum estradiol levels were roughly 7 ng/mL at 36 weeks’ gestation [36] while another study determined this level to be 20 ng/mL during the third trimester [37]. During the postpartum period, serum estradiol levels were reported as 20 pg/mL at week 1 and increased slightly to above 40 pg/mL after 6 months [38]. If the decline in estrogen levels is as severe as indicated and if there is a lag time for estrogen levels to rebound after birth, this could lead to lower abundances of lactobacilli in vaginal communities for quite some time post-delivery [5, 6]. A limitation of our study is that we did not directly measure estrogen levels to evaluate how this might contribute to changes in vaginal community composition observed in our cohort.

In addition to the decline in estrogen levels, normal physiological changes that occur during labor and delivery could influence the dramatic shift in vaginal community composition. Immediately before and during parturition, innate immune cells are activated to initiate an inflammatory process that promotes uterine contractions, dilation of the cervix, and rupturing of fetal membranes [39]. In our study, vaginal levels of hyaluronan and Hsp70 were significantly elevated in postpartum samples, while both D- and L-lactic acid were substantially lower. Hyaluronan is a high molecular weight glycosaminoglycan that is present in the extracellular matrix [40]. Prior to and during labor, hyaluronan levels increase corresponding to a softening and dilation of the cervix in preparation for delivery [41]. The increased hyaluronan level observed in postpartum samples most likely reflects the consequences of ongoing cervical remodeling after delivery. Although not measured in the current study, we expect the detected increase was in the level of low molecular weight hyaluronan fragments [42]. Elevations in Hsp70 could indicate the occurrence of functional changes in the genital tract as a result of cervical remodeling. Given that both Hsp70 and hyaluronan fragments activate pro-inflammatory immune responses [43–45], the altered environmental conditions would be expected to induce a stress response in the affected cells. This will modify the properties of vaginal epithelial and immune cells leading to changes in the composition of vaginal fluid. Moreover, a decrease in the levels of D- and L-lactic acid reduce their ability to provide multiple protective mechanisms in the vagina including the suppression of pathogens [46–48], promotion of DNA repair in vaginal epithelial cells [49], and enhancement of innate and adaptive immunity [50]. Although we did not measure changes in vaginal pH, it is probable that the lower levels of lactic acid postpartum reduced vaginal acidification leading to a decrease in inhibition of non-lactobacilli proliferation. In our study, the concentrations of L-lactic acid and hyaluronan also correlated with a decrease or increase in ⍺-diversity, respectively. We anticipate this association is simply the byproduct of changes in host physiology during and after parturition. Nonetheless, the combination of higher levels of Hsp70 and hyaluronan and lower levels of lactic acid creates an environment that contributes to the persistence of diverse bacterial communities in the vagina post-delivery.

Avershina et al. determined that changes in vaginal community composition can occur as soon as labor begins [14]. Due to all postpartum samples being obtained approximately 1 month after delivery, we are unable to comment on possible changes that may have occurred immediately after birth or at later periods. However, we have confirmed a marked shift from a *Lactobacillus*-dominated vaginal microbiota to one in which lactobacilli are depleted and replaced by a diverse number of other bacteria that occur during the pregnancy to postpartum transition. Furthermore, these variations in the vaginal microbiota are consequences of alterations in nutrient availability, vaginal pH, and local immune parameters that change the ability of different bacterial species to survive and proliferate.

## Supplementary Information

Fig. S1 Relative proportions of bacteria in the vaginal communities of 48 pregnant women separated by stage. The stacked bars represent the proportions of bacterial taxa within one sample. Bars are separated by the pregnancy stage in which the sample was collected (top headings). Taxa colors are indicated in the legend below the figure. “Other” represents the sum of all bacterial taxa that were not present at 1% in at least two women. (PDF 40 kb)

ESM 2 (XLSX 153 kb)

ESM 3 (DOCX 39 kb)

## Data Availability

The sequence data analyzed in this study along with the corresponding metadata are available in the NCBI Sequence Read Archive under BioProject PRJNA614593 (“Study of the pregnant and postpartum period vaginal microbiome;” http://www.ncbi.nlm.nih.gov/bioproject/614593).
