# Supplementary material for: Changes in the Vaginal Microbiome during the Pregnancy to Postpartum Transition
Source: Reprod Sci. 2021 Jan 11;28(7):1996–2005. doi: 10.1007/s43032-020-00438-6 (PMC8189965; doi:10.1007/s43032-020-00438-6)
Supplement: Supplementary file 1 — Relative proportions of bacteria in the vaginal communities of 48 pregnant women separated by stage. The stacked bars represent the proportions of bacterial taxa within one sample. Bars are separated by the pregnancy stage in which the sample was collected (top headings). Taxa colors are indicated in the legend below the figure. “Other” represents the sum of all bacterial taxa that were not present at 1% in at least two women. (PDF 40 kb) [file 43032_2020_438_MOESM1_ESM.pdf]

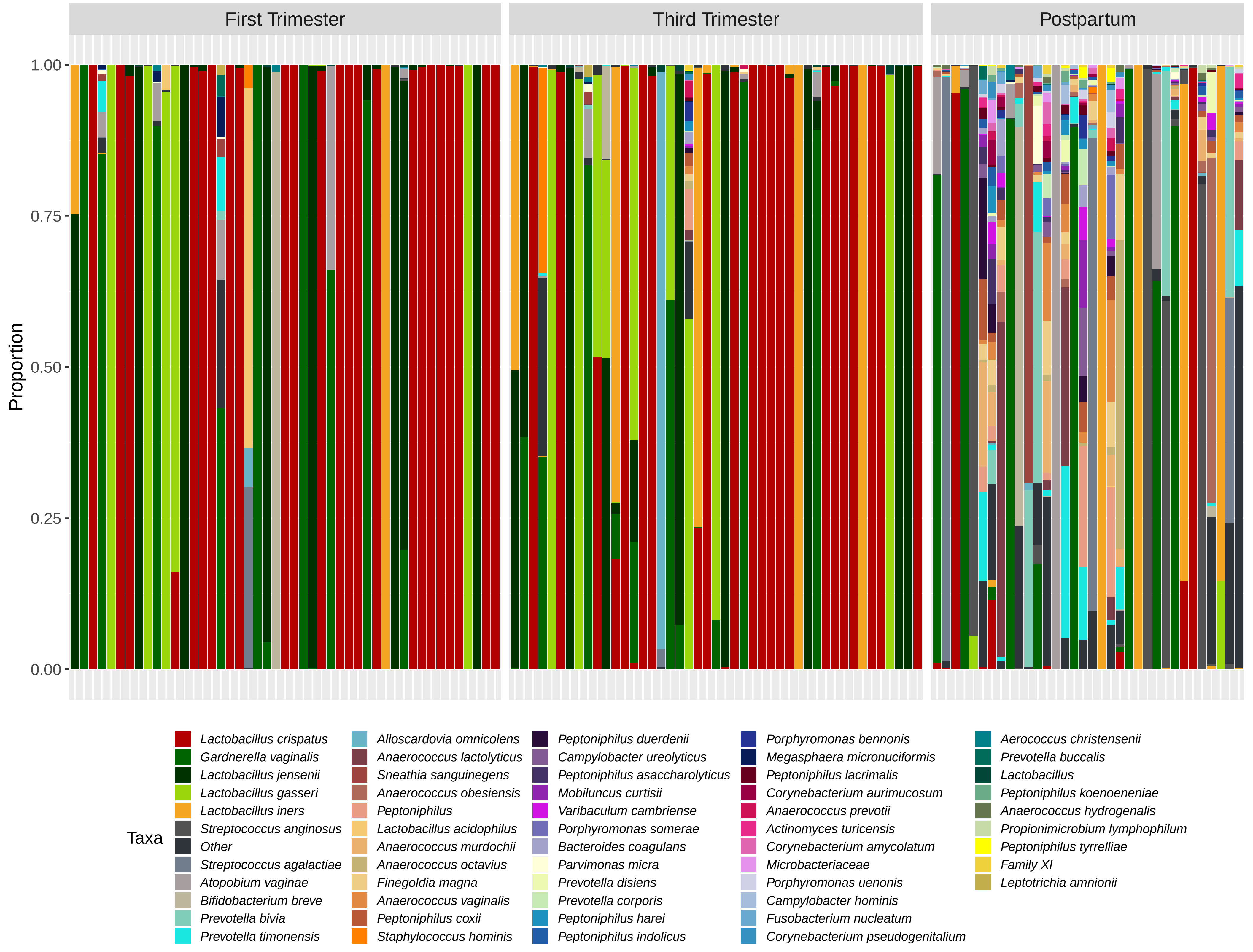
First Trimester
Third Trimester
Postpartum
Proportion
1.00
0.75
0.50
0.25
0.00
Taxa
Lactobacillus crispatus
Gardnerella vaginalis
Lactobacillus jensenii
Lactobacillus gasseri
Lactobacillus iners
Streptococcus anginosus
Other
Streptococcus agalactiae
Atopobium vaginae
Bifidobacterium breve
Prevotella bivia
Prevotella timonensis
Alloscardovia omnicolens
Anaerococcus lactolyticus
Sneathia sanguinegens
Anaerococcus obesiensis
Peptoniphilus
Lactobacillus acidophilus
Anaerococcus murdochii
Anaerococcus octavius
Finegoldia magna
Anaerococcus vaginalis
Peptoniphilus coxii
Staphylococcus hominis
Peptoniphilus duerdenii
Campylobacter ureolyticus
Peptoniphilus asaccharolyticus
Mobiluncus curtisii
Varibaculum cambriense
Porphyromonas somerae
Bacteroides coagulans
Parvimonas micra
Prevotella disiens
Prevotella corporis
Peptoniphilus harei
Peptoniphilus indolicus
Porphyromonas bennonis
Megasphaera micronuciformis
Peptoniphilus lacrimalis
Corynebacterium aurimucosum
Anaerococcus prevotii
Actinomyces turicensis
Corynebacterium amycolatum
Microbacteriaceae
Porphyromonas uenonis
Campylobacter hominis
Fusobacterium nucleatum
Corynebacterium pseudogenitalium
Aerococcus christensenii
Prevotella buccalis
Lactobacillus
Peptoniphilus koenoeneniae
Anaerococcus hydrogenalis
Propionimicrobium lymphophilum
Peptoniphilus tyrrelliae
Family XI
Leptotrichia amnionii
