# Supplementary material for: Changes in the Vaginal Microbiome during the Pregnancy to Postpartum Transition
Source: Reprod Sci. 2021 Jan 11;28(7):1996–2005. doi: 10.1007/s43032-020-00438-6 (PMC8189965; doi:10.1007/s43032-020-00438-6)
Supplement: Supplementary file 3 — (DOCX 39 kb) [file 43032_2020_438_MOESM3_ESM.docx]

**Table S1. Relative proportions of bacterial taxa identified in 48 women from pregnancy to postpartum.** Samples were collected during three stages; A = First Trimester, B = Third Trimester, C = Postpartum. The taxa in the far left column represent all 358 taxa identified in the samples. [.xlsx file]

**Table S2. Rank abundance of the top 10 taxa in each stage**.

|  | **First Trimester** | |  | **Third Trimester** | |  | **Postpartum** | |
| --- | --- | --- | --- | --- | --- | --- | --- | --- |
| **Rank** | **Taxa** | **Count** |  | **Taxa** | **Count** |  | **Taxa** | **Count** |
| 1 | *Lactobacillus crispatus* | 224555 |  | *Lactobacillus crispatus* | 416632 |  | *Gardnerella vaginalis* | 88691 |
| 2 | *Gardnerella vaginalis* | 112324 |  | *Lactobacillus jensenii* | 119300 |  | *Lactobacillus iners* | 52707 |
| 3 | *Lactobacillus jensenii* | 65349 |  | *Lactobacillus gasseri* | 78973 |  | *Streptococcus anginosus* | 52499 |
| 4 | *Lactobacillus gasseri* | 40472 |  | *Lactobacillus iners* | 50426 |  | *Lactobacillus crispatus* | 39129 |
| 5 | *Bifidobacterium breve* | 10284 |  | *Gardnerella vaginalis* | 43474 |  | *Prevotella bivia* | 17412 |
| 6 | *Lactobacillus iners* | 9108 |  | *Alloscardovia omnicolens* | 5606 |  | *Streptococcus agalactiae* | 15220 |
| 7 | *Atopobium vaginae* | 8356 |  | *Staphylococcus hominis* | 3290 |  | *Atopobium vaginae* | 14326 |
| 8 | *Lactobacillus acidophilus* | 3227 |  | *Bifidobacterium breve* | 2159 |  | *Prevotella timonensis* | 13572 |
| 9 | *Prevotella timonensis* | 2841 |  | *Atopobium vaginae* | 1706 |  | *Sneathia sanguinegens* | 13037 |
| 10 | *Lachnospiraceae* | 2096 |  | *Staphylococcus haemolyticus* | 1312 |  | *Anaerococcus lactolyticus* | 12248 |

**Table S3. Dominant^a^ bacterial species in the vaginal communities of study participants at each pregnancy stage sampled.**

| **Subject** | **First Trimester^b,c^** | **Third Trimester** | **Postpartum** |
| --- | --- | --- | --- |
| 49 | *Lactobacillus jensenii* | *Lactobacillus iners* | *Gardnerella vaginalis* |
| 55 | *Gardnerella vaginalis* | *Lactobacillus jensenii* | *Streptococcus agalactiae* |
| 56 | *Lactobacillus crispatus* | *Lactobacillus crispatus* | *Lactobacillus crispatus* |
| 57 | *Gardnerella vaginalis* | Mixed | *Gardnerella vaginalis* |
| 61 | *Lactobacillus gasseri* | *Lactobacillus gasseri* | *Streptococcus anginosus* |
| 63 | *Lactobacillus crispatus* | NA | Mixed |
| 65 | *Lactobacillus crispatus* | *Lactobacillus crispatus* | Mixed |
| 66 | *Lactobacillus jensenii* | *Lactobacillus jensenii* | NA |
| 70 | *Lactobacillus gasseri* | *Lactobacillus gasseri* | *Anaerococcus lactolyticus* |
| 71 | *Gardnerella vaginalis* | *Gardnerella vaginalis* | *Gardnerella vaginalis* |
| 72 | *Lactobacillus gasseri* | NA | NA |
| 74 | *Lactobacillus gasseri* | *Lactobacillus crispatus* | *Bifidobacterium breve* |
| 75 | *Lactobacillus jensenii* | *Lactobacillus jensenii* | *Sneathia sanguinegens* |
| 76 | *Lactobacillus crispatus* | NA | NA |
| 79 | *Lactobacillus crispatus* | *Lactobacillus iners* | Mixed |
| 80 | *Lactobacillus crispatus* | *Lactobacillus crispatus* | Mixed |
| 81 | Mixed | *Lactobacillus gasseri* | NA |
| 82 | *Lactobacillus crispatus* | *Lactobacillus crispatus* | NA |
| 83 | *Lactobacillus crispatus* | *Lactobacillus crispatus* | *Atopobium vaginae* |
| 84 | *Lactobacillus acidophilus* | *Alloscardovia omnicolens* | NA |
| 85 | *Gardnerella vaginalis* | *Gardnerella vaginalis* | Mixed |
| 86 | *Lactobacillus jensenii* | *Lactobacillus jensenii* | *Gardnerella vaginalis* |
| 87 | *Bifidobacterium breve* | *Lactobacillus gasseri* | Mixed |
| 89 | *Lactobacillus crispatus* | *Lactobacillus iners* | *Streptococcus agalactiae* |
| 91 | *Lactobacillus crispatus* | *Lactobacillus crispatus* | *Lactobacillus iners* |
| 92 | *Gardnerella vaginalis* | *Lactobacillus gasseri* | Mixed |
| 93 | *Lactobacillus jensenii* | *Lactobacillus jensenii* | NA |
| 94 | *Lactobacillus crispatus* | *Lactobacillus crispatus* | *Anaerococcus octavius* |
| 95 | *Gardnerella vaginalis* | *Gardnerella vaginalis* | NA |
| 96 | *Lactobacillus crispatus* | *Lactobacillus crispatus* | NA |
| 97 | *Lactobacillus crispatus* | *Lactobacillus crispatus* | NA |
| 98 | *Lactobacillus crispatus* | *Lactobacillus crispatus* | NA |
| 100 | *Gardnerella vaginalis* | *Lactobacillus crispatus* | *Gardnerella vaginalis* |
| 104 | *Lactobacillus crispatus* | *Lactobacillus crispatus* | *Lactobacillus iners* |
| 109 | *Lactobacillus iners* | *Lactobacillus iners* | NA |
| 110 | *Lactobacillus jensenii* | *Lactobacillus jensenii* | *Streptococcus anginosus* |
| 111 | *Lactobacillus jensenii* | *Gardnerella vaginalis* | *Gardnerella vaginalis* |
| 117 | *Lactobacillus crispatus* | *Lactobacillus crispatus* | *Streptococcus anginosus* |
| 118 | *Lactobacillus crispatus* | *Lactobacillus crispatus* | *Gardnerella vaginalis* |
| 121 | *Lactobacillus crispatus* | *Lactobacillus crispatus* | *Lactobacillus iners* |
| 123 | *Lactobacillus crispatus* | *Lactobacillus crispatus* | *Lactobacillus crispatus* |
| 124 | NA | *Lactobacillus iners* | *Streptococcus anginosus* |
| 128 | *Lactobacillus crispatus* | *Lactobacillus crispatus* | *Anaerococcus obesiensis* |
| 130 | *Lactobacillus crispatus* | *Lactobacillus crispatus* | *Lactobacillus iners* |
| 132 | *Lactobacillus gasseri* | *Lactobacillus gasseri* | Mixed |
| 144 | *Lactobacillus jensenii* | *Lactobacillus jensenii* | *Streptococcus* |
| 145 | *Lactobacillus crispatus* | *Lactobacillus jensenii* | NA |
| 157 | *Lactobacillus crispatus* | *Lactobacillus crispatus* | NA |
| a Dominance is defined as having a relative proportion greater than 50% within the community.  b NA = missing data; c Mixed = the community contained multiple species and was not dominated by any one | | | |

**Table S4. Results of linear mixed-effects models of ⍺-diversity including random slopes for subject.**

| Model and Parameters^a^ | Model Summary^b^ | | | |  |
| --- | --- | --- | --- | --- | --- |
| Model 1: Shannon diversity ~ L-lactic acid + Hyaluronan + Stage + (1 \| Subject) + Ɛ | | |  |  |  |
|  |  |  |  |  |  |
| Random effects: |  |  | Number of: | |  |
| Groups | Variance | SD | Observations | Groups |  |
| Subject (Intercept) | 0.012 | 0.109 | 125 | 48 |  |
| Residual | 0.333 | 0.577 |  |  |  |
|  |  |  |  |  |  |
| Fixed effects: | Coefficients | SE | df | P value |  |
| (Intercept) | 0.326 | 0.092 | 119.475 | 5.27E-04 | *** |
| L-lactic acid | -0.142 | 0.058 | 116.168 | 1.49E-02 | * |
| Hyaluronan | 0.283 | 0.070 | 114.766 | 9.34E-05 | *** |
| Stage Third Trimester | 0.073 | 0.124 | 82.956 | 5.56E-01 |  |
| Stage Postpartum | 0.427 | 0.166 | 109.714 | 1.14E-02 | * |
|  |  |  |  |  |  |
| Model 2: Simpson diversity ~ L-lactic acid + Hyaluronan + (1 \| Subject) + Ɛ | | |  |  |  |
|  |  |  |  |  |  |
| Random effects: |  |  | Number of: | |  |
| Groups | Variance | SD | Observations | Groups |  |
| Subject (Intercept) | 0.003 | 0.054 | 125 | 48 |  |
| Residual | 0.046 | 0.213 |  |  |  |
|  |  |  |  |  |  |
| Fixed effects: | Coefficients | SE | df | P value |  |
| (Intercept) | 0.193 | 0.021 | 42.815 | 6.74E-12 | *** |
| L-lactic acid | -0.060 | 0.021 | 120.516 | 5.03E-03 | ** |
| Hyaluronan | 0.135 | 0.021 | 121.686 | 2.14E-09 | *** |
| ^a^ Stage = pregnancy stage; ℇ = random error; fixed effects were chosen using the step() function in the R package lmerTest (v 3.1.0) | | | | | |
| ^b^ SD = standard deviation, SE = standard error; df = degrees of freedom; significance is indicated as follows: "*", P < 0.05; "**", P > 0.01; ***, P < 0.001 | | | | | |
